# Pharmacokinetic interactions of esaxerenone with amlodipine and digoxin in healthy Japanese subjects

**DOI:** 10.1186/s40360-020-00423-4

**Published:** 2020-07-29

**Authors:** Yoshiaki Kirigaya, Masanari Shiramoto, Tomoko Ishizuka, Hinako Uchimaru, Shin Irie, Manabu Kato, Takako Shimizu, Takafumi Nakatsu, Yasuhiro Nishikawa, Hitoshi Ishizuka

**Affiliations:** 1 Daiichi Sankyo Co., Ltd., 1-2-58 Hiromachi, Shinagawa-ku, Tokyo, 140-8710 Japan; 2 SOUSEIKAI Hakata Clinic, 6-18, Tenyamachi, Hakata-ku, Fukuoka, 812-0025 Japan

**Keywords:** Esaxerenone, Drug interactions, Pharmacokinetics, Digoxin, Amlodipine

## Abstract

**Background:**

To investigate the effects of coadministration of esaxerenone with amlodipine on the pharmacokinetics (PK) of each drug, and of esaxerenone on the PK of digoxin.

**Methods:**

In three open-label, single-sequence, crossover studies, healthy Japanese males received single oral doses of esaxerenone 2.5 mg (Days 1, 15), with amlodipine 10 mg/day (Days 8–18) (Study 1, *N* = 24); single doses of amlodipine 2.5 mg (Days 1, 21), with esaxerenone 5 mg/day (Days 8–25) (Study 2; *N* = 20); or digoxin 0.25 mg/day (Days 1–15) with esaxerenone 5 mg/day (Days 11–15) (Study 3; *N* = 20). PK parameters and safety were assessed.

**Results:**

Study 1: esaxerenone peak plasma concentration (C_max_) and time to C_max_ were unaltered by amlodipine coadministration, but mean half-life was slightly prolonged from 18.5 to 20.9 h. Geometric least-squares mean (GLSM) ratios for C_max_, area under the plasma concentration–time curve (AUC) from zero to last measurable concentration and from zero to infinity for esaxerenone + amlodipine versus esaxerenone were 0.958, 1.154, and 1.173, respectively. Study 2: corresponding GLSM ratios for amlodipine + esaxerenone versus amlodipine were 1.099, 1.185, and 1.214. Study 3: esaxerenone did not markedly alter digoxin PK. GLSM ratios for C_max_, trough plasma concentration, and AUC during a dosing interval for digoxin versus esaxerenone + digoxin were 1.130, 1.088, and 1.072, respectively.

**Conclusions:**

No drug–drug interactions are expected during combination therapy with esaxerenone and either amlodipine or digoxin, based on a lack of any clinically relevant PK changes.

**Trial registration:**

Studies 1 and 2: JapicCTI-163379 (registered on 20 September 2016); Study 3: JapicCTI-163443 (registered on 24 November 2016).

## Background

Hypertension is an important public health issue and cardiovascular risk factor [1–3]. Effective control of hypertension can reduce the risk of cardiovascular and cerebrovascular complications and end-organ damage [4–6]. Recent large international population-based surveys have reported optimal control of blood pressure (BP) in only 25–50% of subjects [7–10], and combination antihypertensive therapy is often required to attain such control [11, 12]. In Japan, intensive antihypertensive therapy is required to achieve strict systolic BP (SBP)/diastolic BP (DBP) goals of < 130/80 mmHg, or < 140/90 mmHg in hypertensive patients [13]. The mean number of antihypertensive drugs prescribed for patients in the overall Japanese population was reported as 1.9 (±1.0) between April 2014 and March 2015 [14].

Calcium-channel blockers (CCBs) are the most widely used antihypertensive agents, particularly in Asia, because of their potent BP-lowering abilities [15]. One of the most commonly prescribed CCBs is amlodipine as either mono- or combination therapy [16]. However, since at least half of patients have treatment-resistant hypertension that fails to adequately respond to initial multi-drug therapy, including CCB-containing treatment combinations [7–10], focus has turned to therapeutic agents that exert antihypertensive effects through different mechanisms of action [17]. Mineralocorticoid receptor (MR) blocker exerts their antihypertensive effects through inhibition of ligand binding and activation of MR, which differs from CCBs, and the novel nonsteroidal MR blocker esaxerenone was recently approved for the treatment of hypertension in Japan [18]. In a phase 1 study, esaxerenone exposure after single and multiple doses in healthy volunteers was generally dose-proportional [19]. After multiple daily doses of esaxerenone 10–100 mg for 10 days, time to peak plasma concentration (t_max_) was 2.5–3.5 h and elimination half-life (t_1/2_) was 22.3–25.1 h. In a mass balance study, about one-third of the clearance of esaxerenone was found to be through oxidative metabolism by CYP3A [20].

Amlodipine is mainly metabolised by CYP3A and is a weak *in vivo* inhibitor of CYP3A [21, 22]. In a previous study of healthy Japanese subjects, peak plasma concentration (C_max_) and area under the plasma concentration–time curve (AUC) for midazolam, a CYP3A-index substrate [23, 24], were increased by approximately 20% when coadministered with esaxerenone, which was not a clinically meaningful effect [25]. Therefore, it seems unlikely that there would be any clinically relevant drug–drug interactions (DDIs) between amlodipine and esaxerenone. However, if concurrent use of amlodipine and esaxerenone to treat hypertension is to become widespread, the potential for DDIs between these two agents should be evaluated.

Digoxin is a P-glycoprotein (P-gp) substrate and P-gp plays a major role in both the absorption and elimination of digoxin; thus, P-gp inhibition is a known risk factor for increased digoxin exposure [26, 27]. Although esaxerenone has inhibitory activity against P-gp *in vitro* [28], the effect is not considered to be clinically significant and according to the guidance on drug interaction studies [29], a DDI study is not required. However, given that MR blockers such as esaxerenone may frequently be administered with digoxin, the interaction between these drugs is important and a clinical assessment was merited. This is because digoxin has a narrow therapeutic window [30, 31], and DDI studies with digoxin are recommended by International Council for Harmonisation E7 guidelines [32].

Therefore, the aim of the study was to clarify DDIs between esaxerenone and amlodipine or digoxin, by investigating the effects of esaxerenone and amlodipine coadministration on the pharmacokinetics (PK) of esaxerenone (Study 1) and amlodipine (Study 2), and the effects of esaxerenone on the PK of digoxin (Study 3).

## Methods

### Study design and treatments

**Fig. 1:**
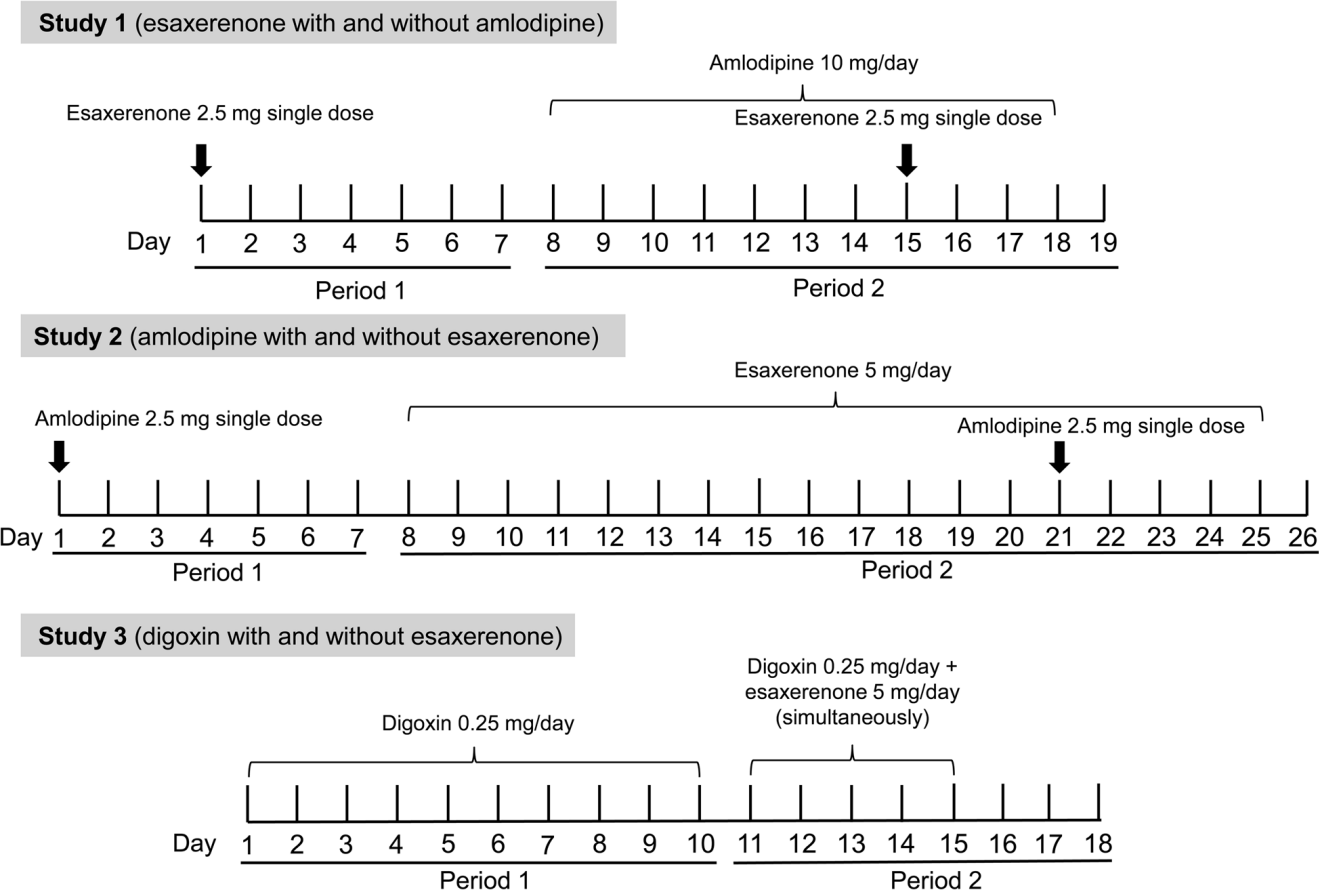
Designs of the pharmacokinetic studies

All studies had a single-centre, open-label, single-sequence design (Fig. 1). All subjects gave written informed consent. Doses, study periods and intervals in each study were designed in accordance with recent DDI study guidelines [23, 24]. The doses of the substrate drugs used were selected from those in the linear PK range. The doses of the perpetrator drugs were selected as the highest daily dose to maximize the possibility of demonstrating a DDI. Study periods were set to achieve PK steady state in the perpetrator drug.

#### Study 1

Period 1 (Days 1–7) comprised the esaxerenone alone single-dose administration phase of the study. A single esaxerenone 2.5-mg tablet (Daiichi Sankyo Co., Ltd., Tokyo, Japan) was administered orally to fasting subjects (Day 1; Period 1). Period 2 (Days 8–19) comprised the coadministration phase of the study, commencing 7 days after the first dose of esaxerenone. On Day 8, amlodipine 10 mg (AMLODIN®; Sumitomo Dainippon Pharma Co., Ltd., Osaka, Japan) was administered orally after breakfast once daily for 11 days. On Day 15, esaxerenone 2.5-mg and amlodipine 10-mg tablets were coadministered orally to subjects in the fasting state (Fig. 1). A final follow-up visit was taken on Days 24–26.

#### Study 2

A similar 2-period study design to Study 1 was implemented for Study 2. In Period 1 (Days 1–7), a single 2.5-mg dose of amlodipine was administered orally after breakfast (Day 1). During Period 2 (Days 8–26), esaxerenone 5 mg was administered orally after breakfast once daily for 18 days. On Day 21, esaxerenone 5 mg and amlodipine 2.5 mg were coadministered orally after breakfast (Fig. 1). A final follow-up visit was taken on Days 31–33.

#### Study 3

This study comprised two periods. In Period 1 (Days 1–10), a 0.25-mg tablet of digoxin (DIGOSIN®; Chugai Pharmaceutical Co., Ltd., Tokyo, Japan) was administered orally once daily after breakfast for 10 days (Days 1–10). There was no washout, and Period 2 started immediately after completion of Period 1. In Period 2 (Days 11–18), a 0.25-mg dose of digoxin and 5-mg dose of esaxerenone were administered orally at the same time each day (in the fed state) for 5 days (Fig. 1). A final follow-up visit was taken on Days 23–25.

Additional details on the treatments in all three studies are provided in Additional file 1**.**

### Study population

The inclusion criteria were the same for all three studies. All studies included healthy Japanese males aged 20–45 years and with a body mass index (BMI) of ≥18.5 to < 25.0 kg/m^2^. All subjects had sitting BP of < 140/90 mmHg, and heart rates of ≤99 beats/min at screening. Details of exclusion criteria are provided in Additional file 1.

### PK assessments

#### Blood sampling

In Study 1, blood samples (3 mL) were collected for drug concentration measurement of esaxerenone on Days 1 and 15, before and at 0.5, 1, 1.5, 2, 2.5, 3, 3.5, 4, 6, 8, 12, 24, 48, 72, and 96 h after esaxerenone administration. In Study 2, blood samples (5 mL) were collected for drug concentration measurement of amlodipine on Days 1 and 21, before and at 2, 3, 4, 5, 6, 7, 8, 9, 12, 24, 48, 72, 96, and 120 h after amlodipine administration. In Study 3, blood samples (3 mL) for drug concentration measurement of digoxin were collected on Days 10 and 15, before and at 0.25, 0.5, 1, 1.5, 2, 3, 4, 6, 8, 12, 16, and 24 h after digoxin administration.

Plasma for assays of esaxerenone, amlodipine, and digoxin was obtained by centrifugation of the blood samples (at 4 °C and 1700×*g* for 10 min) and was subsequently frozen (− 20 °C or lower) until delivered to the laboratory for analysis.

#### Plasma assay

Drug concentrations were measured by liquid chromatography-tandem mass spectrometry (LC-MS/MS). The methodology for chromatographic separation and determination of esaxerenone used in Studies 1 and 2 has been reported previously [19, 33]. For amlodipine, plasma samples were treated by solid phase extraction (OASIS HLB μElution plate, Waters Corporation, Milford, MA, USA), and chromatographic separation was performed using a column (Capcell Pak® C18 MGII, Shiseido, Tokyo, Japan) with an internal diameter of 2.0 mm, a length of 50 mm, and a pore size of 3 μm. Detection was performed using API 5000 (AB SCIEX, Framingham, MA, USA) tandem mass spectrometry with electrospray ionisation (ESI) in the positive detection mode; multiple reaction monitoring (MRM) of amlodipine (m/z 409–238) and its internal standard (amlodipine-d4, m/z 413–238) was conducted. For amlodipine test samples of 0.05, 0.125, 1.25, and 8.0 ng/mL, the intra-study assay precision rates were 2.1, 1.5, 2.2, and 0.7%, respectively. Accuracy of the assay ranged from 4.0 to 11.0%, with a lower limit of quantification (LLOQ) of 0.05 ng/mL.

In Study 3, methods for the determination of esaxerenone plasma concentrations were identical to those used in Studies 1 and 2 (described above). For digoxin, plasma samples were treated by solid phase extraction (ISOLUTE SLE+ 200 mg; Biotage AB, Uppsala, Sweden) and chromatographic separation was performed using a SunShell C18 column (ChromaNik Technologies Inc., Osaka, Japan) with an internal diameter of 2.1 mm, a length of 50 mm, and a pore size of 2.6 μm. Detection was performed using Triple Quad 5500 (AB SCIEX, Framingham, MA, USA) tandem mass spectrometry with ESI in the positive ion mode; MRM of digoxin (m/z 798–651) and its internal standard (digoxin-d3, m/z 801–654) was conducted. For digoxin test samples of 0.05, 0.1, 1.0, and 20.0 ng/mL, the intra-study assay precision rates were 3.3, 5.7, 1.5, and 1.8%, respectively. Accuracy of the assay ranged from − 4.0 to 7.0%, with an LLOQ of 0.05 ng/mL.

#### PK analysis

PK parameters were calculated by non-compartmental analysis, using Phoenix® WinNonlin® (version 6.3; Certara, Princeton, NJ, USA).

For Studies 1 and 2, the primary endpoints were C_max_ and AUC to the last quantifiable time (AUC_last_) and from time zero to infinity (AUC_inf_) for esaxerenone. Secondary endpoints in both studies included time to maximum esaxerenone concentration (t_max_), esaxerenone half-life (t_1/2_), and apparent total body clearance (CL/F).

For Study 3, the primary endpoints were C_max_, trough plasma concentration (C_trough_), and AUC during a dosing interval (AUC_tau_). Secondary endpoints included t_1/2_, t_max_, and apparent total body clearance at steady state (CL_ss_/F).

### Safety

Safety was evaluated through the assessment of adverse events (AEs), laboratory tests, vital signs (BP, pulse rate, and body temperature), and 12-lead electrocardiogram. AEs were coded using Medical Dictionary for Regulatory Activities (MedDRA/J version 19.0, 19.1) System Organ Class and Preferred Terms.

### Sample size

The sample size was calculated assuming within-subject variations in C_max_ and AUC of 20 and 10%, respectively, based on previous studies [33–35]. Assuming that geometric least-squares mean (GLSM) ratios of C_max_ and AUC were ≤ 1.05, when ratios were estimated after a single oral dose of test drug (esaxerenone, amlodipine, or digoxin) and concomitant drug administration, a sample size of 18 subjects would provide ≥80% statistical power with two-sided 90% confidence intervals (CIs) for GLSM ratios of C_max_ and AUC to detect the CIs within 0.80–1.25. To allow for unexpected circumstances, such as subject withdrawals, the number of subjects was specified as 24 in Study 1 and as 20 in Studies 2 and 3.

### Statistical analyses

In all studies, the PK analysis sets included subjects who received test drugs (esaxerenone, amlodipine, or digoxin), and for whom data were available for at least one primary endpoint in Periods 1 and 2. The safety analysis sets included all subjects who agreed to participate in the study and who received at least a dose of drug (esaxerenone, amlodipine, or digoxin). Differences in PK parameters between treatment groups were calculated by ratios of GLSM and their 90% CIs. No apparent DDI was concluded if the GLSM ratio was contained within the bounds (0.80–1.25) of 90% CIs. In all statistical analyses, SAS (version 9.2; SAS Institute, Cary, NC, USA) was used.

## Results

### Baseline characteristics

Baseline characteristics for subjects in all three studies are shown in Table 1. Twenty-four subjects were enrolled into Study 1; two withdrew, one each due to an AE and subject decision, and were not included in the analysis. In Study 2, a total of 20 subjects were enrolled and two withdrew due to an AE or subject decision and were not included in the analysis. In Study 3, a total of 20 subjects were enrolled. One individual withdrew due to an AE and was not included in the analysis.

**Table 1:** Demographic characteristics of study subjects at baseline (PK analysis set)

Characteristic	Study 1 (***n*** = 22)	Study 2 (***n*** = 18^a^)	Study 3 (***n*** = 19^a^)
Age, years	27.2 ± 6.2	31.4 ± 7.4	28.5 ± 8.4
Height, cm	171.09 ± 4.55	171.12 ± 6.03	170.58 ± 5.79
Weight, kg	62.69 ± 6.91	64.98 ± 7.56	62.73 ± 6.30
Body mass index, kg/m^2^	21.40 ± 2.08	22.04 ± 1.63	21.56 ± 1.78

Values are mean ± standard deviation

*PK* pharmacokinetic

^a^Subjects who were withdrawn were not included in this analysis

### Effect of amlodipine on esaxerenone PK (Study 1)

**Fig. 2:**
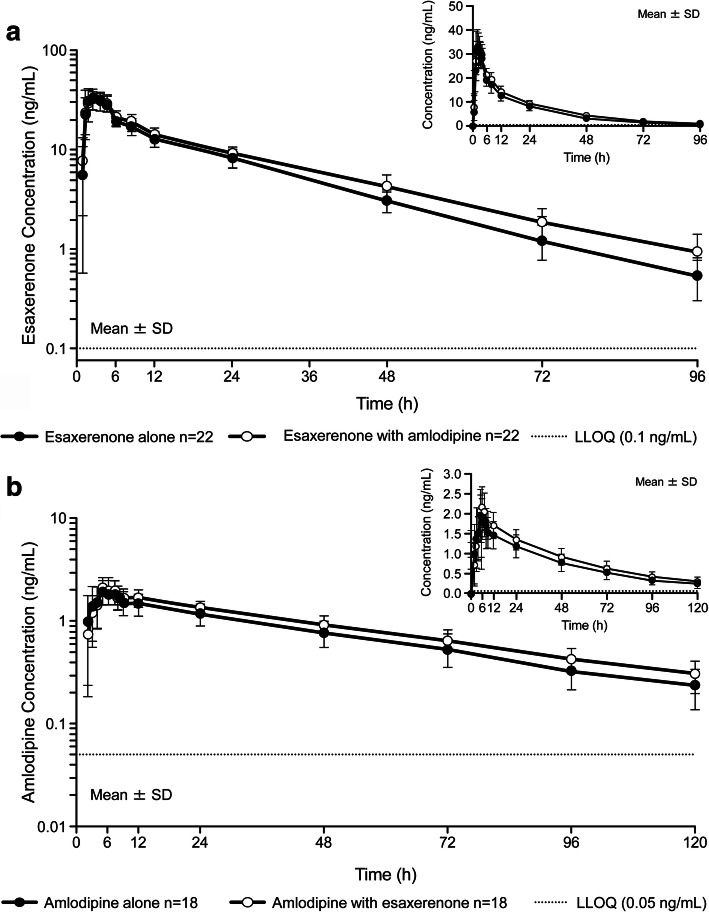
Plasma concentration–time profiles for Studies 1 and 2. Healthy Japanese males were administered either **a**) esaxerenone alone and with amlodipine (Study 1) or **b**) amlodipine alone and with esaxerenone (Study 2). Both panels show semi-log plots with linear plots as insets. LLOQ, lower limit of quantification; SD, standard deviation

Esaxerenone plasma concentration–time profiles, alone and in combination with amlodipine, are shown in Fig. 2a. Esaxerenone C_max_ and t_max_ did not differ but AUC_last_ and AUC_inf_ were slightly increased when coadministered with amlodipine (Table 2). The mean t_1/2_ for esaxerenone was slightly prolonged from 18.5 to 20.9 h when esaxerenone was coadministered with amlodipine (Table 2). GLSM ratios (90% CI) for C_max_, AUC_last_, and AUC_inf_ for esaxerenone plus amlodipine versus esaxerenone alone were 0.958 (0.905–1.015), 1.154 (1.118–1.190), and 1.173 (1.136–1.212), respectively (Table 3).

**Table 2:** Pharmacokinetic parameters for each drug alone and in combination

Parameter	Study 1		Study 2		Study 3	
	Esaxerenone 2.5 mg		Amlodipine 2.5 mg		Digoxin 0.25 mg/day	
	Alone (***n*** = 22)	+ Amlodipine 10 mg/day (***n*** = 22)	Alone (***n*** = 18)	+ Esaxerenone 5 mg/day (***n*** = 18)	Alone (***n*** = 19)	+ Esaxerenone 5 mg/day (***n*** = 19)
C_max_, ng/mL	35.5 ± 6.3	33.9 ± 5.0	2.09 ± 0.47	2.28 ± 0.46	1.54 ± 0.53	1.77 ± 0.73
C_trough_, ng/mL	–	–	–	–	0.533 ± 0.089	0.583 ± 0.116
AUC_last_, ng·h/mL	560 ± 106	644 ± 111	87.2 ± 22.3	102 ± 20	–	–
AUC_inf_, ng·h/mL	575 ± 111	674 ± 127	102 ± 29	122 ± 29	–	–
AUC_tau_, ng·h/mL	–	–	–	–	15.3 ± 2.5	16.5 ± 3.3
t_max_, h^a^	2.00 (1.00–4.00)	2.50 (1.50–4.00)	5.00 (3.00–9.00)	6.00 (5.00–7.00)	1.50 (0.50–4.00)	1.00 (0.50–3.00)
t_1/2_, h	18.5 ± 3.2	20.9 ± 3.1	40.5 ± 6.8	43.5 ± 6.7	NA	NA
CL/F, L/h	4.49 ± 0.79	3.83 ± 0.67	26.8 ± 8.9	21.8 ± 5.9	–	–
CL_ss_/F, L/h	–	–	–	–	16.8 ± 3.2	15.8 ± 3.5

Unless stated otherwise, values are means±standard deviations

NA, not assessable because the elimination rate constant was not appropriately estimated

*AUC*_*inf*_ area under the plasma concentration–time curve up to infinity, *AUC*_*last*_ AUC up to the last quantifiable time, *AUC*_*tau*_ AUC over the dosing interval, *CL/F* apparent total body clearance, *CL*_*ss*_*/F* apparent total body clearance at steady state, *C*_*max*_ peak plasma concentration, *C*_*trough*_ trough plasma concentration, *t*_*1/2*_ terminal elimination half-life, *t*_*max*_ time to reach maximum plasma concentration

^a^Median value (range)

**Table 3:** Pharmacokinetic parameters and ratios based on geometric least squares means

	Treatment group		
**Study 1**	**Esaxerenone (*n*** **= 22)**	**+ Amlodipine (*n*** **= 22)**	**Ratio (90% CI)**
C_max_, ng/mL	35.0	33.5	0.958 (0.905, 1.015)
AUC_last_, ng·h/mL	551	635	1.154 (1.118, 1.190)
AUC_inf_, ng·h/mL	565	663	1.173 (1.136, 1.212)
**Study 2**	**Amlodipine (*n*** **= 18)**	**+ Esaxerenone (*n*** **= 18)**	**Ratio (90% CI)**
C_max_, ng/mL	2.04	2.24	1.099 (1.059, 1.140)
AUC_last_, ng·h/mL	84.4	100	1.185 (1.132, 1.240)
AUC_inf_, ng·h/mL	97.6	118	1.214 (1.157, 1.273)
**Study 3**	**Digoxin (*n*** **= 19)**	**+ Esaxerenone (*n*** **= 19)**	**Ratio (90% CI)**
C_max_, ng/mL	1.47	1.66	1.130 (0.998, 1.280)
C_trough_, ng/mL	0.526	0.572	1.088 (1.033, 1.145)
AUC_tau_, ng·h/mL	15.1	16.2	1.072 (1.015, 1.133)

*AUC*_*inf*_ area under the plasma concentration–time curve up to infinity, *AUC*_*last*_ AUC up to the last quantifiable time, *AUC*_*tau*_ AUC over the dosing interval, *CI* confidence interval, *C*_*max*_ peak plasma concentration, *C*_*trough*_ trough plasma concentration

### Effect of esaxerenone on amlodipine PK (Study 2)

Amlodipine plasma concentrations, alone and in combination with esaxerenone, are shown in Fig. 2b. The C_max_ of amlodipine was slightly increased when amlodipine was coadministered with esaxerenone (Table 2). The t_max_ of amlodipine was unaffected by coadministration with esaxerenone. Amlodipine AUC_last_ and AUC_inf_ were slightly increased, as was amlodipine t_1/2_ (from 40.5 to 43.5 h), when amlodipine was coadministered with esaxerenone (Table 2). GLSM ratios (90% CI) for C_max_, AUC_last_, and AUC_inf_ for amlodipine plus esaxerenone versus amlodipine alone were 1.099 (1.059–1.140), 1.185 (1.132–1.240), and 1.214 (1.157–1.273), respectively (Table 3).

### Effect of esaxerenone on digoxin PK (Study 3)

**Fig. 3:**
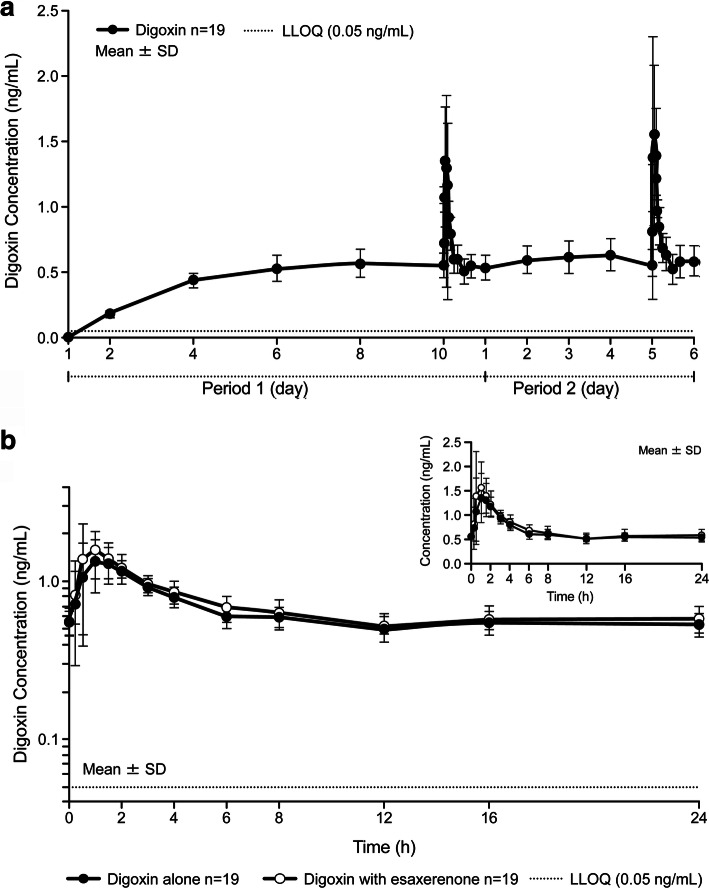
Plasma concentration–time profiles for Study 3. Healthy Japanese males for Study 3 showing the following: **a**) changes in digoxin concentration and **b**) digoxin alone and in combination with esaxerenone; a semi-log plot with a linear plot as an inset. LLOQ, lower limit of quantification; SD, standard deviation

Trough plasma concentrations (C_trough_) of digoxin reached steady state after Day 6 (Fig. 3a). Digoxin plasma concentrations, alone and in combination with esaxerenone are shown in Fig. 3b. The digoxin C_max_ was slightly increased when digoxin was coadministered with esaxerenone (Table 2). The digoxin AUC_tau_ increased slightly when the drug was coadministered with esaxerenone. GLSM ratios (90% CI) for C_max_, C_trough_, and AUC_tau_ for digoxin alone versus esaxerenone plus digoxin were 1.130 (0.998–1.280), 1.088 (1.033–1.145), and 1.072 (1.015–1.133), respectively (Table 3).

### Safety

A summary of AEs in all three studies is provided in Additional file 2**,** Supplementary Table S1. No deaths or serious AEs occurred. In Study 1, two subjects reported treatment-emergent AEs (TEAEs), including decreased appetite and gastroenteritis (*n* = 1; esaxerenone alone), and muscle spasms and increased creatine phosphokinase levels (*n* = 1; esaxerenone with amlodipine). Both events were of mild severity and resolved without treatment, but the subject with gastroenteritis discontinued study medication. No TEAEs were considered by the investigators to have a causal relationship with treatment. In Study 2, only one AE occurred (a case of tonsillitis 2 days after the single dose of amlodipine). This was of moderate severity, resolved with drug therapy, and was considered unrelated to study treatment. In Study 3, one subject had nasopharyngitis and one had increased levels of alanine aminotransferase and aspartate aminotransferase during treatment with digoxin alone; the AEs were mild in severity and resolved without treatment. No TEAEs occurred during coadministration of digoxin and esaxerenone.

## Discussion

Data from these three studies indicate no clinically relevant DDIs or safety concerns associated with concurrent dosing of esaxerenone with either amlodipine or digoxin.

Regarding a potential effect of amlodipine on esaxerenone PK, 90% CI values for GLSM ratios for C_max_ and AUC of esaxerenone with amlodipine versus esaxerenone alone were within the range 0.80–1.25, indicating that esaxerenone PK parameters were not affected by amlodipine. In contrast, evaluation of the effect of esaxerenone on amlodipine PK revealed increases of approximately 20% in the AUC for amlodipine. Given that an AUC increase of 60% was observed when amlodipine was coadministered with a moderate CYP3A4 inhibitor (diltiazem) [36], and the prescribing information for amlodipine states that amlodipine should be used with caution when used together with moderate or strong CYP3A4 inhibitors [21], the AUC increase of 20% observed in the current analysis was considered to be not clinically significant.

When digoxin was coadministered with esaxerenone, the digoxin C_max_ increased by approximately 13%. Although prior *in vitro* data revealed that esaxerenone had inhibitory activity against P-gp [28], the inhibition was weak, and an *in vivo* DDI study with a P-gp probe substrate was not deemed necessary, based on available guidance for DDI studies [29]. In this investigation, although the digoxin C_max_ increased slightly when digoxin was coadministered with esaxerenone, other parameters (including AUC_tau_) were within predefined ranges. Digoxin prescribing information states that dose adjustment is recommended when an increase in AUC is ≥14% [37]. Therefore, we conclude that esaxerenone had no clinically relevant impact on the steady-state PK of digoxin.

There were no safety issues when a single dose of esaxerenone 2.5 mg was coadministered with multiple doses of amlodipine 10 mg/day, a single dose of amlodipine 2.5 mg was coadministered with multiple doses of esaxerenone 5 mg/day, or when esaxerenone 5 mg was coadministered with digoxin 0.25 mg/day.

The main limitation of these studies was that they were designed to evaluate PK parameters in healthy subjects and the efficacy and safety of the treatment combinations were not evaluated in patients. Although no notable safety concerns were raised in our analyses, assessment of long-term administration is warranted to confirm the detailed safety profile associated with concurrent dosing.

## Conclusions

The PK of esaxerenone were unaffected by coadministration of amlodipine. Although slight increases in amlodipine and digoxin C_max_ were observed during coadministration of esaxerenone, these alterations were not considered clinically relevant. No safety concerns were seen when amlodipine or digoxin was coadministered with esaxerenone. These findings indicate that, from a PK standpoint, no significant dosage adjustment is necessary for amlodipine or digoxin when administered with esaxerenone in hypertensive patients requiring combination therapy.

## Supplementary information

**Additional file 1.** Additional methods describing details on treatments and exclusion criteria.

**Additional file 2: Table S1.** Summary of adverse events.

## Data Availability

All aggregate data relevant to this analysis are included in this article. Additional de-identified subject data and supporting documents pertaining to these studies, such as the study protocol and statistical analysis plan, are provided upon reasonable request made via this web address (https://vivli.org/ourmember/daiichi-sankyo/) in accordance with the data sharing policy of Daiichi Sankyo Co., Ltd.

## Abbreviations

AE: Adverse event
AUC: Area under the plasma concentration-time curve
AUC_inf_: Area under the plasma concentration-time curve from time zero to infinity
AUC_last_: Area under the plasma concentration-time curve to the last quantifiable time
AUC_tau_: Area under the plasma concentration-time curve during a dosing interval
BMI: Body mass index
BP: Blood pressure
CCB: Calcium-channel blocker
CI: Confidence interval
CL/F: Apparent total body clearance
CL_ss_/F: Apparent total body clearance at steady state
C_max_: Peak plasma concentration
C_trough_: Trough plasma concentration
DBP: Diastolic blood pressure
DDI: Drug–drug interactions
ESI: Electrospray ionisation
GLSM: Geometric least-squares mean
LC-MS/MS: Liquid chromatography-tandem mass spectrometry
LLOQ: Lower limit of quantification
MR: Mineralocorticoid receptor
MRM: Multiple reaction monitoring
P-gp: P-glycoprotein
PK: Pharmacokinetic
SBP: Systolic blood pressure
t_1/2_: Elimination half-life
t_max_: Maximum concentration
