# Supplementary material for: Pharmacokinetic interactions of esaxerenone with amlodipine and digoxin in healthy Japanese subjects
Source: BMC Pharmacol Toxicol. 2020 Jul 29;21:55. doi: 10.1186/s40360-020-00423-4 (PMC7389645; doi:10.1186/s40360-020-00423-4)
Supplement: Supplementary file 1 — Additional file 1. Additional methods describing details on treatments and exclusion criteria. [file 40360_2020_423_MOESM1_ESM.docx]

***BMC Pharmacology and Toxicology***

**Additional file 1**

**Pharmacokinetic interactions of esaxerenone with amlodipine and digoxin in healthy Japanese subjects**

Yoshiaki Kirigaya^1^, Masanari Shiramoto^2^, Tomoko Ishizuka^1^, Hinako Uchimaru^2^, Shin Irie^2^, Manabu Kato^1^, Takako Shimizu^1^, Takafumi Nakatsu^1^, Yasuhiro Nishikawa^1^and Hitoshi Ishizuka^1^

^1^Daiichi Sankyo Co., Ltd., 1-2-58 Hiromachi, Shinagawa-ku, Tokyo 140-8710, Japan.

^2^SOUSEIKAI Hakata Clinic, 6-18, Tenyamachi, Hakata-ku, Fukuoka 812-0025, Japan.

**Corresponding author:**

Yoshiaki Kirigaya

Clinical Pharmacology Department, Daiichi Sankyo Co., Ltd., 1-2-58 Hiromachi, Shinagawa-ku, Tokyo 140-8710, Japan.

Telephone: +81-8010136896

Fax: +81-357403625

Email: kirigaya.yoshiaki.c8@daiichisankyo.co.jp

# Methods

***Additional details on treatments***

In all three studies, study drugs were administered orally with 200 mL of water. No other beverages were permitted for 1 hour before or 2 hours after administration. Caffeinated drinks were prohibited during hospitalisation, and subjects were only permitted food prepared by the study centre, which was provided at predetermined times. Following drug administration on Days 1 and 15 (Study 1), Days 1 and 21 (Study 2), or Days 10 and 15 (Study 3), subjects rested in a seated position for 4 hours.

***Subject exclusion criteria***

Exclusion criteria included the following: any previous serious disease affecting central nervous, cardiovascular, respiratory, haematopoietic, gastrointestinal, hepatic, thyroid, pituitary, or adrenal systems or organs; hypersensitivity to drugs, including amlodipine, or idiosyncratic reactions (e.g. penicillin allergy); drug or alcohol dependence; a positive infection test result (hepatitis B surface antigen, hepatitis C virus antibody, syphilis, or human immunodeficiency virus antibody); collection of ≥1200 mL of whole blood within 1 year, ≥400 mL of whole blood within 84 days, or ≥200 mL of whole blood within 28 days, before screening; plasmapheresis or platelet apheresis within 14 days before screening; participation in another clinical study and receipt of a study drug within 120 days before screening; previous participation in a clinical study of esaxerenone; inability to use contraception during the study; use of any drugs or supplements (including food and supplements containing St John’s wort) inducing cytochrome P450 (CYP)3A4 within 30 days before study drug administration; likely to require any drug other than the study drug after screening until the end of study; use of concomitant therapy within 30 days before study drug administration; clinically problematic subjective symptoms (e.g. headache, dizziness, sleepiness, and feelings of weakness) or objective findings (e.g. decreased BP); electrocardiograph abnormalities; laboratory test abnormalities; ingestion of grapefruit (juice or pulp) within 7 days before hospital admission; and individuals considered by the investigator or subinvestigator to be ineligible for participation in the study (e.g. expected difficulties with study visits or drug compliance).
