# Supplementary material for: Pharmacokinetic interactions of esaxerenone with amlodipine and digoxin in healthy Japanese subjects
Source: BMC Pharmacol Toxicol. 2020 Jul 29;21:55. doi: 10.1186/s40360-020-00423-4 (PMC7389645; doi:10.1186/s40360-020-00423-4)
Supplement: Supplementary file 2 — Additional file 2: Table S1. Summary of adverse events. [file 40360_2020_423_MOESM2_ESM.docx]

***BMC Pharmacology and Toxicology***

**Additional file 2**

**Pharmacokinetic interactions of esaxerenone with amlodipine and digoxin in healthy Japanese subjects**

Yoshiaki Kirigaya^1^, Masanari Shiramoto^2^, Tomoko Ishizuka^1^, Hinako Uchimaru^2^, Shin Irie^2^, Manabu Kato^1^, Takako Shimizu^1^, Takafumi Nakatsu^1^, Yasuhiro Nishikawa^1^and Hitoshi Ishizuka^1^

^1^Daiichi Sankyo Co., Ltd., 1-2-58 Hiromachi, Shinagawa-ku, Tokyo 140-8710, Japan.

^2^SOUSEIKAI Hakata Clinic, 6-18, Tenyamachi, Hakata-ku, Fukuoka 812-0025, Japan.

**Corresponding author:**

Yoshiaki Kirigaya

Clinical Pharmacology Department, Daiichi Sankyo Co., Ltd., 1-2-58 Hiromachi, Shinagawa-ku, Tokyo 140-8710, Japan.

Telephone: +81-8010136896

Fax: +81-357403625

Email: kirigaya.yoshiaki.c8@daiichisankyo.co.jp

***Supplementary Table S1.*** *Summary of adverse events*

| **Adverse event** | **Study 1** | | **Study 2** | | **Study 3** | |
| --- | --- | --- | --- | --- | --- | --- |
|  | **Esaxerenone 2.5 mg** | | **Amlodipine 2.5 mg** | | **Digoxin 0.25 mg/day** | |
|  | **Alone (n=24)** | **+ Amlodipine**  **10 mg/day (n=22^a^)** | **Alone (n=20)** | **+ Esaxerenone**  **5 mg/day (n=18^a^)** | **Alone (n=20)** | **+ Esaxerenone 5 mg/day (n=19^a^)** |
|  |  |  |  |  |  |  |
| Any TEAE | 1 (4.2) | 1 (4.5) | 1 (5.0) | 0 | 2 (10.0) | 0 |
| Gastroenteritis | 1 (4.2) | 0 | 0 | 0 | 0 | 0 |
| Tonsillitis | 0 | 0 | 1 (5.0) | 0 | 0 | 0 |
| Nasopharyngitis | 0 | 0 | 0 | 0 | 1 (5.0) | 0 |
| Loss of appetite | 1 (4.2) | 0 | 0 | 0 | 0 | 0 |
| Muscle cramp | 0 | 1 (4.5) | 0 | 0 | 0 | 0 |
| Increased alanine aminotransferase | 0 | 0 | 0 | 0 | 1 (5.0) | 0 |
| Increased aspartate aminotransferase | 0 | 0 | 0 | 0 | 1 (5.0) | 0 |
| Increased blood creatine phosphokinase | 0 | 1 (4.5) | 0 | 0 | 0 | 0 |

Values are number of subjects (%).

^a^Subjects who were withdrawn were not included in this analysis.

TEAE, treatment-emergent adverse event.
